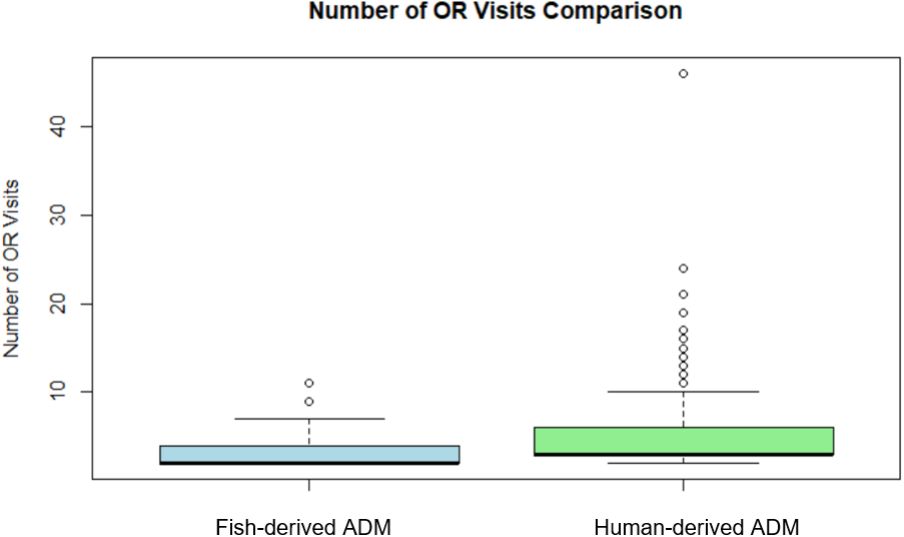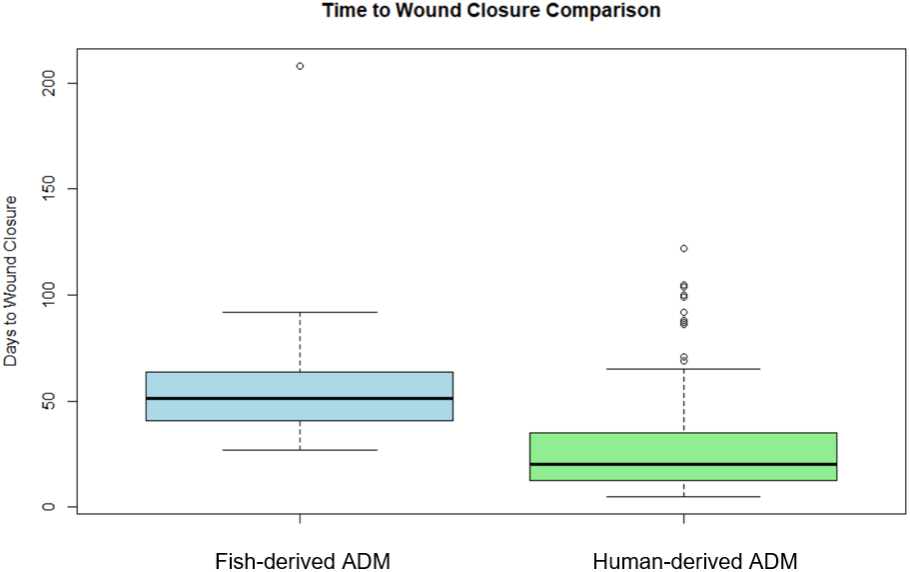# 833 Cost-Effectiveness and Outcomes of Fish- vs. Human-Derived Dermal Matrices in Burn Care

**DOI:** 10.1093/jbcr/iraf019.364

**Published:** 2025-04-01

**Authors:** Anh-Tho Antoinette Nguyen, Derek Bell

**Affiliations:** Kessler Burn Center; Kessler Burn Center

## Abstract

**Introduction:**

Acellular dermal matrices (ADM) are widely used in burn care to enhance wound healing while minimizing the need for repeated surgical interventions. This study compares the clinical outcomes and cost-effectiveness of a fish-derived acellular dermal matrix and a human-derived dermal matrix in burn patients, focusing on surgical efficiency, time to wound closure, complication rates, and hospital length of stay.

**Methods:**

A retrospective cohort study was conducted at an American Burn Association (ABA) verified burn center on 156 burn patients, 18 treated with a fish-derived matrix and 138 with a human-derived matrix. Key outcomes included time to wound closure, number of OR visits, days in hospital, and complication rates. Patients were categorized by race, and statistical analyses were performed using two-sample t-tests, ANOVA, and Tukey’s HSD post-hoc tests.

**Results:**

The fish-derived matrix group required significantly fewer OR visits (mean 3.76 ± 1.25) compared to the human-derived matrix group (mean 5.78 ± 1.57), with a t-value of -2.39 and a p-value of 0.023. However, time to wound closure was longer in the fish-derived matrix group (mean 72.4 ± 16.3 days) compared to the human-derived matrix group (mean 58.7 ± 12.9 days), with a t-value of 2.12 and a p-value of 0.045. Hospital length of stay was not significantly different between the groups (fish-derived: 23.4 ± 8.7 days, human-derived: 21.1 ± 7.9 days, p = 0.32). Complication rates were similar (fish-derived: 16.7%, human-derived: 15.9%; p = 0.89).

Despite the longer time to wound closure in the fish-derived matrix group, the reduction in OR visits resulted in estimated per-patient savings of $4,040 to $9,090 based on typical OR visit costs.

Race was not a significant factor influencing time to wound closure in the fish-derived matrix group (ANOVA, p = 0.945), though a significant trend toward longer healing times was found in Asian patients treated with the human-derived matrix (p = 0.049).

**Conclusions:**

In burn patients, the fish-derived acellular dermal matrix demonstrated greater surgical efficiency with fewer OR visits, potentially leading to significant cost savings despite a longer time to wound closure. These findings support the use of the fish-derived matrix in cases where reducing the frequency of surgical interventions is key.

**Applicability of Research to Practice:**

The findings suggest that fish-derived acellular dermal matrices reduce surgical interventions, offering a cost-effective alternative to human-derived matrices in burn care. With fewer OR visits and comparable outcomes in complication rates and hospital stays, the fish-derived matrix presents an efficient option for improving patient care and reducing healthcare costs, especially in resource-limited settings.

**Funding for the Study:**

N/A